# 

**Published:** 1865-05

**Authors:** 


BOOKS AND PAMPHLETS RECEIVED.

*A Vest-Pocket Medical Lexicon; being a Dictionary of Words, Terms, and Symbols of Medical Science, collated from the best authorities, with the addition of new words not before introduced into a Lexicon, with an Appendix.* BY D. B. ST. JOHN ROOSA, M. D., *Aural Surgeon to the New York Eye and Ear Infirmary. New York:* WM. WOOD & CO., 61 *Walker Street*, 1865.

*An Address delivered at the Inauguration of the Dale General Hospital, U. S. A.* BY WARREN WEBSTER, M. D., *Assistant Surgeon U. S. Army, in charge of De Camp General Hospital, Davids' Island, New York Harbor.*

*A Sermon on the Death of Abner; preached in the John Street Presbyterian Church, Belleville, C. W.* BY WM. MCLAREN.

